# Structure of the 2,4′-dihydroxyacetophenone dioxygenase from *Alcaligenes* sp. 4HAP

**DOI:** 10.1107/S1399004714015053

**Published:** 2014-08-29

**Authors:** R. Keegan, A. Lebedev, P. Erskine, J. Guo, S. P. Wood, D. J. Hopper, S. E. J. Rigby, J. B. Cooper

**Affiliations:** a STFC Rutherford Appleton Laboratory, RAL, Harwell Oxford, Didcot OX11 0FA, England; b Laboratory of Protein Crystallography, Centre for Amyloidosis and Acute Phase Proteins, UCL Division of Medicine (Royal Free Campus), Rowland Hill Street, London NW3 2PF, England; c Institute of Biological, Environmental and Rural Sciences, Aberystwyth University, Penglais, Aberystwyth SY23 3DA, Wales; d Faculty of Life Sciences, Manchester Institute of Biotechnology, University of Manchester, 131 Princess Street, Manchester M1 7DN, England

**Keywords:** dioxygenase, cupin fold, iron binding, catalytic mechanism

## Abstract

The enzyme 2,4′-dihydroxyacetophenone dioxygenase (DAD) catalyses the conversion of 2,4′-dihydroxyacetophenone to 4-hydroxybenzoic acid and formic acid with the incorporation of molecular oxygen. Whilst the vast majority of dioxygenases cleave within the aromatic ring of the substrate, DAD is very unusual in that it is involved in C—C bond cleavage in a substituent of the aromatic ring. There is evidence that the enzyme is a homotetramer of 20.3 kDa subunits, each containing nonhaem iron, and its sequence suggests that it belongs to the cupin family of dioxygenases. In this paper, the first X-ray structure of a DAD enzyme from the Gram-negative bacterium *Alcaligenes* sp. 4HAP is reported, at a resolution of 2.2 Å. The structure establishes that the enzyme adopts a cupin fold, forming dimers with a pronounced hydrophobic interface between the monomers. The catalytic iron is coordinated by three histidine residues (76, 78 and 114) within a buried active-site cavity. The iron also appears to be tightly coordinated by an additional ligand which was putatively assigned as a carbonate dianion since this fits the electron density optimally, although it might also be the product formate. The modelled carbonate is located in a position which is highly likely to be occupied by the α-hydroxyketone group of the bound substrate during catalysis. Modelling of a substrate molecule in this position indicates that it will interact with many conserved amino acids in the predominantly hydrophobic active-site pocket where it undergoes peroxide radical-mediated heterolysis.

## Abstract 2

The first X-ray structure of a 2,4′-dihydroxyacetophenone dioxygenase from *Alcaligenes* sp. 4HAP at a resolution of 2.2 Å is reported. This structure establishes that the enzyme adopts the cupin-fold, forming compact dimers with a pronounced hydrophobic interface between the monomers. Each monomer possesses a catalytic ferrous iron that is coordinated by three histidines (76, 78 and 114) and an additional ligand which has been putatively assigned as a carbonate, although formate and acetate are possibilities.

## 1. Introduction   

**Figure 1:**
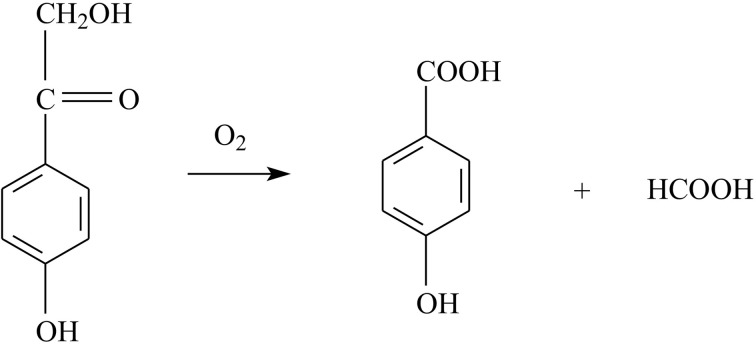
The reaction catalysed by 2,4′-dihydroxyacetophenone dioxygenase (DAD). The enzyme has a high affinity for oxygen, which is used for C—C bond cleavage of the substrate (2,4′-dihydroxyacetophenone), yielding 4-hydroxybenzoic acid and formate.

The enzyme 2,4′-dihydroxyacetophenone dioxygenase (DAD) catalyses the conversion of 2,4′-dihydroxyaceto­phenone (2,4′-DHAP), a breakdown product of lignin, to 4-hydroxy­benzoic acid and formic acid with the incorporation of molecular oxygen (Fig. 1 ▶). As a bacterial dioxygenase, DAD plays an important environmental role in the aerobic catabolism of aromatic compounds (Gibson & Parales, 2000 ▶; Vaillancourt *et al.*, 2006 ▶). The expression of enzymes such as DAD in appropriately engineered microorganisms has potential for the removal of aromatic pollutants from soil and groundwater and in the production of novel chemicals, for example in biotransformations (Bugg *et al.*, 2011 ▶). Indeed, the bacterial species from which the enzyme originates (*Alcaligenes*) has been used industrially for biotransformations in the production of unusual amino acids, polyhydroxybutanol and polyhydroxybutyrates for use in new biodegradable plastics (see, for example, Nair *et al.*, 2009 ▶).

Whilst the vast majority of dioxygenases cleave within the aromatic ring, DAD is very unusual in that it is involved in C—C bond cleavage in a substituent of the aromatic ring. The DAD enzyme from *Alcaligenes* sp. 4HAP has been cloned and expressed in *Escherichia coli* (Hopper & Kaderbhai, 1999 ▶). Each subunit of the DAD enzyme consists of 177 amino acids and has a molecular mass of 20.3 kDa. The DAD enzyme contains nonhaem iron which prior to this work was of unknown redox state and is not lost during prolonged dialysis, suggesting that it is tightly bound. There is evidence that the enzyme is a homotetramer of subunits and its sequence suggests that it belongs to the cupin family of dioxygenases, which have β-sheet structures and are known to play key biological roles in metabolism, biosynthesis, oxygen sensing and DNA repair (Chavez *et al.*, 2011 ▶; Dunwell *et al.*, 2004 ▶; Fetzner, 2012 ▶). The biochemical properties of the *Burkholderia* sp. AZ11 enzyme have been studied in detail recently and sequence comparisons have suggested that a number of conserved histidine and carboxylate residues may be important for iron binding (Enya *et al.*, 2012 ▶).

The DAD enzyme is involved in the bacterial breakdown of 4-hydroxyacetophenone (Hopper *et al.*, 1985 ▶). This compound in turn is an intermediate in the degradation of 4-ethylphenol (Darby *et al.*, 1987 ▶), an intermediate that occurs in the breakdown of a multitude of naturally occurring aromatic compounds that are found mainly as plant products, but also as manmade compounds that are either deliberately or accidentally released by the chemical industry. The most abundant of the plant products is lignin, which constitutes between 18 and 35% of the dry weight of plant material (see, for example, Novaes *et al.*, 2010 ▶). The ability to break down lignin, which has a polyaromatic structure, and other aromatic plant products is a unique feature of microorganisms that offers enormous potential for exploitation as a source of organic compounds. Whilst Enya *et al.* (2012 ▶) have pointed out that 2,4′-DHAP occurs in aromatic glycosides of some plants and that DAD may have evolved in soil bacteria to break these substances down, they also suggest that the enzyme may have an as yet unknown role in the metabolism of other substances.

Oxygenases such as DAD are by definition enzymes that catalyse reactions in which O atoms are incorporated into aromatic substrates to facilitate their metabolism. This was demonstrated in isotope experiments using ^18^O_2_, where the products of the reaction were shown to contain labelled oxygen (Hayaishi, 1962 ▶, 1966 ▶). The oxygenases fall into two classes, namely the monooxygenases and dioxygenases, which differ in the number of oxygen equivalents that are incorporated. Generally, the metabolism of aromatic compounds involves ring opening, which usually proceeds by the incorporation of at least two hydroxyl groups into the ring either *ortho* or *para* to each other. The ring is then cleaved either at the C—C bond between the two hydroxyls (catalysed by an intradiol dioxygenase) or at an adjacent C—C bond (catalysed by an extradiol dioxygenase). To break the bonds in aliphatic substituents of the ring, or in aliphatic compounds in general, lyase and hydrolase reactions are commonly used in nature, although sometimes monooxygenases are involved. Thus, whilst the vast majority of dioxygenases cleave within the aromatic nucleus, the enzyme in this study (DAD) is one of the few that are involved in C—C bond cleavage in a substituent of the aromatic ring (see Fig. 1 ▶). As with the ring-opening enzymes, isotope experiments using ^18^O_2_ have confirmed that the DAD enzyme is a true dioxygenase, *i.e.* both of the atoms of O_2_ are incorporated into the substrate stoichiometrically (Hopper, 1986 ▶).

Since relatively little is known about the structure and function of this rare class of dioxygenases, we have pursued structural studies of the DAD enzyme as a basis for analysing its mechanism of action. In this work, we have obtained crystals of *Alcaligenes* sp. 4HAP DAD in a form which diffracted synchrotron radiation to 2.2 Å resolution and allowed the first X-ray structure of a DAD enzyme to be determined.

## 2. Methods   

The *Alcaligenes* sp. 4HAP DAD gene was expressed in *E. coli* BL21(DE3) cells using a pET-15b (Novagen) construct to confer the enzyme with a His tag and allow nickel-affinity purification. The yield of affinity-purified protein was estimated to be 20 mg per litre of *E. coli* culture. Following elution from the nickel-affinity column, the protein was buffer-exchanged into 50 m*M* Tris buffer pH 7.5 with 100 m*M* NaCl and 1 m*M* β-mercaptoethanol to lower the imidazole concentration to below 50 m*M*. To obtain crystals of sufficient size for synchrotron X-ray data collection, it was necessary to incubate the purified enzyme at a concentration of 5 mg ml^−1^ with chymotrypsin, which was added in a 1:50 mass ratio prior to setting up hanging-drop crystallization trials following the procedures of Dong *et al.* (2007 ▶) and Wernimont & Edwards (2009 ▶). No efforts were made to specifically remove the His tag with thrombin. The crystal which yielded the data set reported here was grown by the hanging-drop method in 10%(*w*/*v*) PEG 1K, 10% PEG 8K (Molecular Dimensions Structure Screen 2 condition 46). Crystals were cryoprotected by slowly increasing the glycerol concentration to 30%(*v*/*v*) over a period of 1–5 min and were mounted in loops for flash-cooling with an Oxford Cryosystems cryostream. Being very thin needles that were prone to distortion, the majority of the crystals diffracted synchrotron radiation to at best 2.5–3.0 Å resolution with a high rate of radiation damage, particularly on high-intensity beamlines. However, after numerous trials, one crystal eventually yielded 90° of data to a resolution of 2.2 Å at the ESRF, Grenoble, France using the bending-magnet beamline BM14 equipped with a MAR CCD detector. The data were processed with *MOSFLM* (Leslie, 2006 ▶; Powell *et al.*, 2013 ▶) and *SCALA* (Evans, 2006 ▶) in the *CCP*4 suite (Winn *et al.*, 2011 ▶), which suggested that the space group was either *P*6_1_22 or *P*6_5_22, with unit-cell parameters *a* = *b* = 82.6, *c* = 114.0 Å. Data-processing statistics are shown in Table 1 ▶, where it can be seen that the traditional *R*_merge_ is slightly high at 13.6% and the outer shell completeness is slightly low, but that the data are of excellent consistency to 2.2 Å resolution according to the half-set correlation criterion of Karplus & Diederichs (2012 ▶). A more comprehensive account of the expression, purification, crystallization and data collection is given in Beaven *et al.* (2014 ▶).

The structure of DAD was solved with this data set by molecular replacement using an ensemble search model based on residues 35–161 of chain *A* of the RmlC-like cupin protein (PDB entry 3ebr) that was determined by the JCSG (http://www.jcsg.org) and has 29.9% sequence identity to the corresponding fragment of the target sequence. The *A* chains of PDB entries 2o1q (residues 19–144), 3bal (residues 21–149) and 3cjx (residues 21–149) were also used to make up the rest of the ensemble search model. The model was generated by the automated *CCP*4 *BALBES* web service (Long *et al.*, 2008 ▶) and received the best *Q*-factor of 0.635 among all the models generated in the automated search. The low level of the *Q*-factor suggested that further confirmation of the potential solution was needed. Therefore, this ensemble search model was taken from the *BALBES* results and manually put through *MOLREP* (Vagin & Teplyakov, 2010 ▶) in the following space groups: *P*622, *P*6_1_22, *P*6_2_22, *P*6_3_22, *P*6_4_22 and *P*6_5_22. Space group *P*6_5_22 gave the best solution, which was identified after jelly-body refinement of the positioned model with *REFMAC* (Murshudov *et al.*, 1997 ▶, 2011 ▶). This gave *R* and *R*_free_ values of 44.9 and 49.9% compared with the second best solution which had an *R* factor of 51.4% and an *R*_free_ of 54.0% in *P*6_1_22. Subsequently, 25 cycles of automated model building in *ARP*/*wARP* (Langer *et al.*, 2008 ▶) verified that the MR solution was clear and unambiguous, giving an *R* and *R*_free_ of 28.6 and 35.9%, respectively. Further refinement was carried out using the *PDB_REDO* web service (Joosten *et al.*, 2012 ▶), which improved the *R* factor and *R*_free_ to 23.6 and 30.8%, respectively, before modelling was continued in *Coot* (Emsley & Cowtan, 2004 ▶). Figures of the structure were prepared using *CueMol* (http://www.cuemol.org/en) and *POV-Ray* (http://www.povray.org). The solvent-accessible surface and electrostatic potentials were calculated using the *MSMS* (Sanner *et al.*, 1996 ▶) and *APBS* (Baker *et al.*, 2001 ▶) software, respectively. Lattice contacts were analysed by use of the *PISA* server (Krissinel & Henrick, 2007 ▶). The coordinates and structure factors for *Alcaligenes* 2,4′-dihydroxyacetophenone dioxygenase have been deposited in the Protein Data Bank with accession code 4p9g.

Continuous-wave electron paramagnetic resonance (EPR) spectra were obtained from cryofrozen DAD samples at the X band (∼9.4 GHz) using a Bruker ELEXSYS E500 spectrometer equipped with a Super High Q resonator (Bruker ER-4123-SHQ). Temperature control was effected *via* an Oxford Instruments ESR900 helium-flow cryostat interfaced with an ITC503 temperature controller from the same manufacturer. Aliquots of 250 µl DAD (without the His tag) at a concentration of 100 µ*M* were frozen in liquid nitrogen in Wilmad 4 mm outer diameter quartz EPR tubes. For samples analysed with the substrate present, 2,4′-DHAP was added in fivefold molar excess over the protein concentration from a 25 m*M* stock in DMSO. As a control, the addition of an equivalent volume of DMSO lacking the substrate of the protein was shown to have no effect on the EPR spectrum.

## 3. Results   

### 3.1. Crystallographic data   

**Figure 2:**
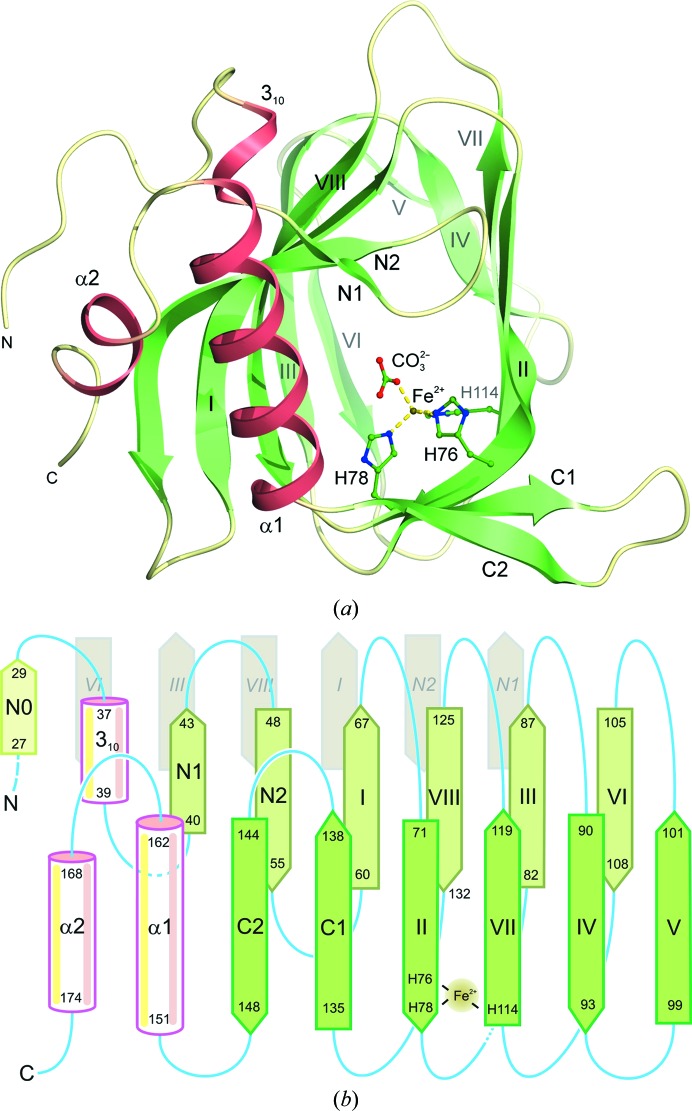
The tertiary structure of *Alcaligenes* DAD. (*a*) The fold of the monomer viewed along the cupin barrel of the enzyme towards the iron-binding site which is formed by three invariant histidine residues (76, 78 and 114) and a putative carbonate ligand (shown in ball-and-stick representation with the iron coloured rust brown and its dative bonds as yellow dashes). Regions of β-strand are coloured pale green and the helical segments are shown in clay-red with the intervening loops in beige. Note that strand N0 partakes in one of the cupin-barrel sheets of the neighbouring monomer in the dimer. (*b*) A topology diagram of DAD showing the cupin-barrel sheets with residue numbers for all of the secondary-structure elements. The iron-binding site formed by His76, His78 and His114 in strands II and VII is shown, as is the involvement of strand N0 in the abutting β-sheet of the neighbouring monomer (pale grey stands at the back, labelled in italics).

Crystals of *Alcaligenes* sp. 4HAP DAD which diffracted synchrotron radiation to 2.2 Å resolution were obtained by *in situ* chymotrypsinolysis (Beaven *et al.*, 2014 ▶). They belonged to space group *P*6_5_22 with one molecule per asymmetric unit and allowed the structure to be solved and refined to an *R* factor of 17.3% and an *R*_free_ of 21.8%. The resulting model of 150 amino acids of the enzyme monomer (Fig. 2 ▶) was found to have 93.6% of the amino acids within the favoured regions of the Ramachandran plot and 98.1% within the allowed areas by the *MolProbity* criteria (Chen *et al.*, 2010 ▶). The electron density for the refined model at 2.2 Å resolution is of very high quality except for the N-terminal 26 amino acids of the protein, which appear to be disordered or may have been cleaved during crystallization. In contrast, no residues are missing at the C-terminal end of the protein. The structure has an estimated r.m.s. coordinate error (Read, 1986 ▶) of 0.18 Å, suggesting that it is defined with high accuracy. Indeed, this is the first structure of a DAD enzyme that has been analysed by X-ray diffraction to date.

### 3.2. Overall structure   

**Figure 3:**
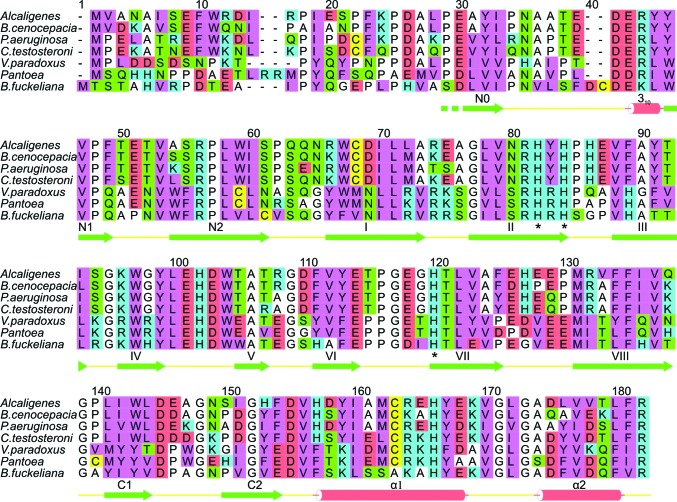
A sequence alignment of putative DAD enzymes. The secondary structure of the *Alcaligenes* sp. 4HAP enzyme is shown below the sequences. The amino-acid residues are colour-coded as follows: cyan, basic; red, acidic; green, neutral polar; pink, bulky hydrophobic; white, Gly, Ala and Pro; yellow, Cys. The iron-binding histidine residues are indicated with asterisks, which also conveniently mark the two cupin motifs (Dunwell *et al.*, 2004 ▶). Numbering refers to the combined alignment.

The protein is a member of the cupin superfamily, which are proteins consisting of a conserved β-barrel fold that are named after the Latin word *cupa*, meaning a barrel. The first members of the cupin family were identified in plants; many are iron-dependent dioxygenases, but some rely on other metals such as nickel or copper for catalytic activity (Dunwell *et al.*, 2004 ▶). Residues 60–132 of DAD form the core cupin fold which consists of a double-stranded β-helix forming two four-stranded antiparallel β-sheets that are a characteristic feature of this superfamily (Fig. 2 ▶). Interestingly, in DAD both of the sheets are actually six-stranded owing to additional β-hairpins formed by residues 40–55 at the N-terminal end and residues 135–148 at the C-terminal end of the cupin motif. Although these additional β-hairpins are at the same end of the cupin barrel and could potentially interact with each other, thus closing this end of the barrel, instead they appear to splay apart and expose an unusual hydrophobic patch formed by Phe43, Trp137, Phe148 and Met156. Movement of these hydrophobic side chains could allow an incoming substrate to access the active site of the enzyme, which is buried inside the cupin barrel. Indeed, the side chain of Met156 appears to be somewhat disordered and has been refined in two conformations. The β-hairpin at the N-terminal end of the cupin barrel is preceded by a short irregular region commencing at the first residue that is visible in the current electron-density map: Ala27. The equivalent C-terminal β-hairpin is followed by two helical segments α1 and α2 (residues 151–162 and 168–174, respectively), which form a helical hairpin that precedes the C-terminal end of the protein. Whilst the electron density is visible right up to the C-terminus, the first 26 amino acids at the N-terminal end of the protein are not defined by the electron-density map at all, suggesting that they are either disordered or have been removed by the chymotrypsin that was essential for crystallization. These N-terminal 25 or so residues seem to be the least conserved amino acids amongst the DADs (Fig. 3 ▶), which is perhaps in accord with their lack of electron density. The remaining secondary-structure elements are well defined by the 2.2 Å resolution map, and the overall tertiary structure of the molecule is shown in Fig. 2 ▶.

Starting at the N-terminal end, there is a short region of β-strand conformation (residues 27–29 or strand N0) followed by an irregular region and then a short 3_10_-helix (residues 37–39), all of which involve reasonably conserved amino acids. This region of 3_10_-helix leads into a well defined β-strand (strand N1), following which there is a sharp β-hairpin turn leading into a larger strand (strand N2) that precedes another β-hairpin turn that then leads into the first strand of the core cupin β-helix domain. There are eight strands making up this domain, which are labelled I–VIII using the notation of Aik *et al.* (2012 ▶) for 2-oxoglutarate oxygenases. The cupin-domain strands form a barrel-like structure made from two antiparallel β-sheets; one of the sheets is formed by strands I, VIII, III and VI, while the other is formed by strands II, VII, IV and V (topology shown in Fig. 2 ▶ *b*). The cupin domain is followed by another β-hairpin formed by strands C1 and C2, which is followed by a helical hairpin formed by helices α1 and α2. The β-hairpin elements at the N- and C-termini of the cupin domain are located at the same end of the cupin barrel in three dimensions and associate with the cupin-barrel strands to give two six-stranded β-sheets. Thus, the first of these sheets is comprised of strands N1, N2, I, VIII, III and VI, while the other is formed by strands C2, C1, II, VII, IV and V, with the N1 and C2 strands forming the outermost lip of the barrel.

An alignment of a selection of proteins sharing sequence identities with the *Alcaligenes* DAD that range from 40 to 90% is shown in Fig. 3 ▶. This demonstrates the marked conservation of these family members, particularly within the strand regions that make up the cupin barrel (strands I–VIII) where the iron-binding histidine residues reside. Strand II contains the first cupin sequence motif (as described by Dunwell *et al.*, 2004 ▶), which has the consensus G(*X*)_3_RH*X*HP and contributes two histidines to the iron-binding site. The second cupin motif of DAD in strand VII has the consensus EGHTLV, in which the sole histidine residue is the third and final amino acid to coordinate the active-site iron. There are several other highly conserved polar residues in DAD, including Asp36, Glu37, Arg38, Arg50, Glu95, His96, Glu97, Glu112, Asp139, Asp149 and His160, which are suggestive of an important role in the folding, stability or catalytic ability of the protein. Inspection of these residues in the structure shows that most of them are solvent-exposed and are involved in ionic interactions with other conserved polar residues and are not directly involved in substrate binding. From the current structure it also does not appear that these charged residues play a major role in quaternary contacts between the DAD monomers. The two cysteines present within DAD (residues 62 and 157) are clearly reduced with their side chains in buried hydrophobic environments and again are unlikely to play a direct role in substrate binding or catalysis.

### 3.3. Quaternary structure   

**Figure 4:**
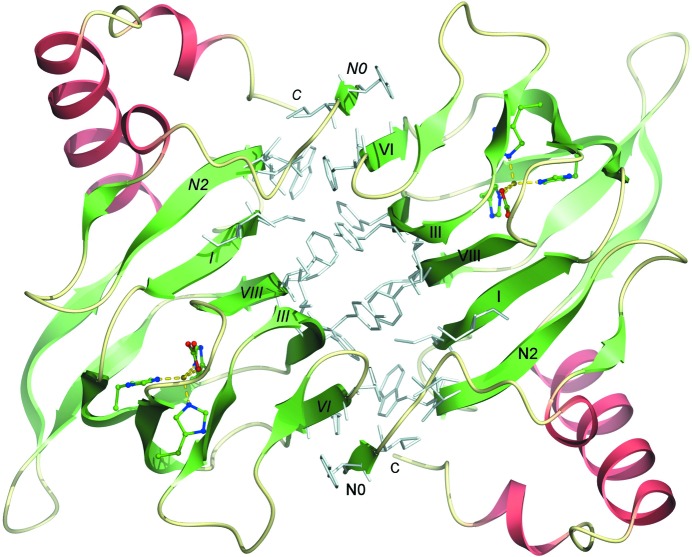
The structure of the DAD dimer. The dimer is viewed along the crystallographic twofold axis which relates the two monomers. The hydrophobic amino acids which form the central dimer interface are shown in pale grey. The secondary-structure elements forming this central β-barrel of the dimer, in which strand N0 crosses over from one subunit to the next, are labelled in roman and italic text to aid in distinguishing the two constituent monomers.

In the crystal structure of DAD, pairs of monomers associate together with an intervening crystallographic twofold such that a substantial hydrophobic interface is formed between them (Fig. 4 ▶). The interface involves five of the six β-strands in the first of the sheets described above, namely strands N2, I, VIII, III and VI of both monomers related by the twofold. The axes of the strands in these two dimer-forming sheets are oriented approximately parallel to one another owing to the fact that they are also roughly parallel to the intervening twofold axis. There are numerous aromatic residues involved in this quaternary interface, including Trp53, Phe83, Tyr85, Phe105, Tyr107 and Phe128, while other hydrophobic residues include Leu52, Cys62, Ile64, Ile87 and Ile130. Interestingly, the extreme N-terminal region of the molecule contains a number of hydrophobic residues which contribute to this interface, namely Tyr28, Ile29 and Ala32. All of the amino acids at this interface are strongly conserved, suggesting that it is a common feature of DAD and its homologues. One important and intriguing feature of the dimerization interface is that residues 28–30 form a very short region of β-strand (strand N0) which takes part in the dimer-forming β-sheet of the cupin barrel of the adjacent subunit. Indeed, we chose this zero-based numbering for the strands at the N-terminus to indicate that the first strand (N0) takes part in a β-sheet belonging to the other monomer. A total of four β-sheet hydrogen bonds are formed by this strand with strand VI of the neighbouring monomer, thus extending the barrel sheet formed by strands N1, N2, I, VIII, III and VI in each monomer of the dimer (see Fig. 2 ▶ *b*).

Analysis using the *PISA* server (Krissinel & Henrick, 2007 ▶) establishes that this interface buries 1182.3 Å^2^ of surface-accessible area of each monomer, which represents 16% of the total surface-accessible area. The interface has a calculated free energy of formation of −17.0 kcal mol^−1^ and involves a total of 18 hydrogen bonds along with four salt bridges. Calculation of the electrostatic surface of the structure establishes that the dimer interface is appreciably more electroneutral than the remainder of the protein surface, which has a relatively uniform negative potential, almost certainly owing to the predominance of acidic amino acids (Asp and Glu) in the sequence, which outnumber the basic residues (Arg and Lys) by a factor of two (22 *versus* 11).

Given that the N-terminal amino acids preceding the first residue of strand N0 (Ala27) are missing from the electron-density map, potentially owing to the action of chymotrypsin during crystallization, the intermolecular β-sheet interactions may actually be more extensive in the full-length protein. Accordingly, in a number of other mono-cupin dioxygenases and homologues of unknown function the N-terminal region performs a similar structural role by interacting with the neighbouring monomer, although not always making a β-sheet interaction with it. The extensive nature of these N-terminal regions suggests that they may be important for oligomerization of the DAD family members. It is conceivable that the lack of the first 26 amino acids in the chymotrypsinolysed protein has affected its stability and/or oligomerization state, perhaps accounting for the observation of dimers in the crystal in contrast to the tetramers observed by analytical ultracentrifugation and gel filtration of the full-length protein in solution (Enya *et al.*, 2012 ▶; Hopper & Kaderbhai, 1999 ▶). However, we confirmed by Superdex 75 gel filtration that the chymotrypsinolysed enzyme also forms tetramers in solution, but following longer incubations with chymotrypsin for several days DAD aggregates further and elutes at the exclusion limit of the column. This suggests that the protein is inherently prone to forming a range of oligomeric states and that perhaps the precipitants needed for crystallization dis­favour the tetramer. We should also mention that the chymotrypsinolysed protein has a comparable catalytic activity to the wild-type enzyme when assayed by the method of Hopper & Kaderbhai (1999 ▶). This suggests that the limited proteolysis has not perturbed the enzyme beyond possibly affecting its quaternary structure in the crystalline state.

In the crystal structure, there is an additional putative dimer interface involving strands II, IV and VII of two monomers related by a crystallographic twofold axis that is different from that generating the main dimer interface. There are a number of buried hydrophobic interactions at this interface formed by Leu94, Val117 and Phe119. Interestingly, these residues are reasonably conserved and are moderately close to the iron-binding site, suggesting that it has some physiological relevance even though the buried surface area per monomer of 711 Å^2^ is rather borderline and this interface does not generate a symmetric tetramer. Hence, these residues might conceivably be involved in interactions with the missing N-terminal portion in higher order quaternary structures, such as the tetramer which the enzyme forms in solution. An alternative, and slightly more plausible, model of the DAD tetramer may be generated by inspection of the three-dimensional structure of the closest homologue of the enzyme, namely the RmlC-like cupin protein from *Ralstonia eutropha* (PDB entry 3ebr). The monomers of this protein associate tightly to form dimers in exactly the same manner as DAD and pairs of these dimers then associate base-to-base *via* the loops linking the strands at the bottom of the cupin barrel in the view shown in Fig. 2 ▶(*b*). The sequences of these loops are quite highly conserved and some residues are invariant, no doubt partly owing to their role in maintaining the cupin fold in the vicinity of the iron-binding site. Hence, the same dimer–dimer interface, which has an intervening twofold axis, may exist in the DAD tetramers, but since the amino acids that are likely to be involved are more polar than those at the monomer–monomer interface in the dimer and the contact area is lower, the tetramer may have lower stability than the dimer.

### 3.4. Active site   

**Figure 5:**
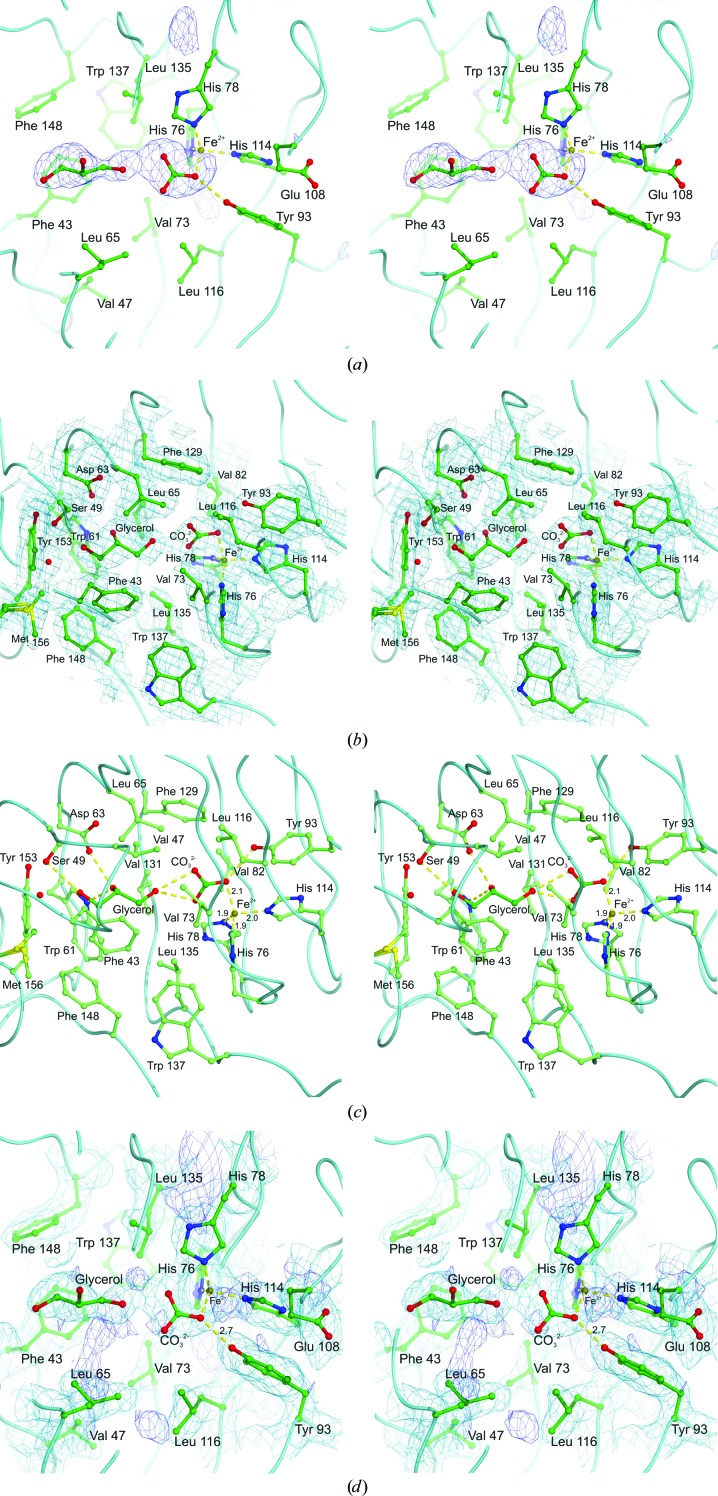
The substrate-binding pocket of DAD. (*a*) A stereoview of the omit map for the active-site region of the protein showing the positive difference electron density for the unknown ligand(s) contoured at 3.0 r.m.s. Our interpretation of this density as an iron-bound carbonate and a cryoprotectant molecule, glycerol, is shown along with the iron-binding histidines and other residues in the vicinity. A more complete view of the active-site residues and putative ligands with their refined electron density, contoured at 1.0 r.m.s., is shown in (*b*). Both ligands fit the electron density satisfactorily, make reasonable hydrogen bonds and refine reasonably well. The dative bonds to the iron ion and the hydrogen bonds made by the putative glycerol and carbonate moieties (dark green) are shown as yellow dashed lines in (*c*). The glycerol makes hydrogen bonds to the putative carbonate and to Ser49, Trp61 and Asp63, which are conserved residues. (*d*) shows more details of the iron ligands along with the electron density for the putative carbonate and glycerol. The refined 2*F*_o_ − *F*_c_ map is shown in pale blue contoured at the same level as in (*b*) and the residual *F*_o_ − *F*_c_ positive difference density, contoured at 2.5 r.m.s., is shown in dark blue. There is a feature of difference density connected with the iron and pointing in the direction of Glu108 which may represent the binding site of diatomic oxygen. Where shown, dative-bond or hydrogen-bond lengths are in Å.

The active site of each monomer is located in a marked hollow in the centre of the barrel which is lined mainly by hydrophobic residues: Phe43, Val47, Trp61, Leu65, Val73, Val82, Tyr93, Leu116, Phe129, Val131, Leu135, Trp137, Phe148, Tyr153 and Met156 (Fig. 5 ▶). These residues have a high level of conservation and some appear to be invariant in the DAD family members shown in Fig. 3 ▶. The catalytic iron is bound at one end of this hollow by three histidine residues (His76, His78 and His114) and what appears to be a carbonate moiety bound to the iron. One O atom of the putative carbonate is approximately 2.0 Å from the iron, suggesting that it is datively bound. The same O atom is within hydrogen-bonding distance of the side chain of Tyr93 which, like the iron-binding histidines, is an invariant feature of DADs. We tested a number of possible ligands that might have been acquired by the protein during expression and purification, including imidazole, which is perhaps the most likely candidate given its presence at high concentrations during the affinity purification. However, these did not fit the electron density or refine as well, and imidazole also makes unfavourable contacts with both the iron and Tyr93. Instead, carbonate does fit well, has a high natural abundance and is known to be a metal ligand in a number of nonhaem iron proteins such as transferrin and lactoferrin (MacGillivray *et al.*, 1998 ▶) as well as a substitute ligand in some dioxygenase mutants (see, for example, Frazee *et al.*, 1998 ▶). Another strong possibility is the reaction product formate, although refinement of this resulted in significant positive difference density (five times the r.m.s.) for the additional O atom that is present in carbonate.

Analysis of DAD sequences allowed Enya *et al.* (2012 ▶) to correctly predict which histidine residues are involved in iron binding on the basis of an alignment with known cupin family members. In addition, they predicted that one of two conserved glutamate residues (Glu95 and Glu108 in *Alcaligenes* sp. 4HAP) might also be involved. However, our X-ray structure reveals that both of these glutamates are on the surface of the protein and, along with a number of highly conserved residues, appear to stabilize the fold of the monomer at the base of the cupin barrel in the vicinity of the β-hairpin loop between strands IV and V.

Whilst an iron bound by three histidine ligands might be expected to confer the protein with a colour, the crystals that we obtained appeared to be colourless, although the purified protein has a grey hue, as was also reported for the *Burkholderia* enzyme (Enya *et al.*, 2012 ▶). It is well known that a number of dioxygenases are colourless. In general for aromatic ring-cleavage enzymes, *ortho*-cleavage (intradiol) Fe^3+^ enzymes are a red colour, whereas *meta*-cleavage (extradiol) Fe^2+^ enzymes are colourless. This tentatively suggests that DAD contains ferrous iron.

The other end of the active-site cavity possesses a polar pocket formed by Tyr153, Ser49 and the main chain of Val41, all of which hydrogen-bond to a single water. These residues are less well conserved than the others mentioned so far, suggesting that some of the weaker DAD homologues (*e.g.* the bottom three sequences shown in Fig. 3 ▶) may have different substrate specificity. The only other polar patch in the cavity is formed by residues Trp61 and Asp63, which are closer to the iron, suggesting that they may be more closely involved in binding polar atoms of the substrate. Of these two residues, the Trp appears to be more conserved than the Asp in evolution.

As mentioned earlier, the presence of a small feature of connected electron density in the active-site cavity suggests that it is occupied by a ligand or possibly by a mixture of ligands which interact with the iron or with the iron-bound carbonate/formate (Fig. 5 ▶ *a*). After several efforts to build in and refine a number of possible compounds that might have been acquired during expression, purification or crystallization, we decided that the most likely candidate was a glycerol molecule bound adjacent to the carbonate. This is feasible since glycerol was used in high concentrations as a cryoprotectant [30%(*v*/*v*)]. Given that the crystals are small, thin needles (typically only 10 µm in width), it is conceivable that the cryoprotectant could diffuse into the active site even though it is somewhat occluded in the crystal structure. The two residues forming the polar patch in the middle of the active-site cavity (Trp61 and Asp63) both form excellent hydrogen bonds to the central hydroxyl group of the putative glycerol moiety. The glycerol hydroxyl group which is closest to the iron forms two hydrogen bonds to the carbonate O atoms and the hydroxyl furthest from the iron makes a weak hydrogen bond to Ser49 at the far end of the pocket.

### 3.5. EPR spectroscopy   

**Figure 6:**
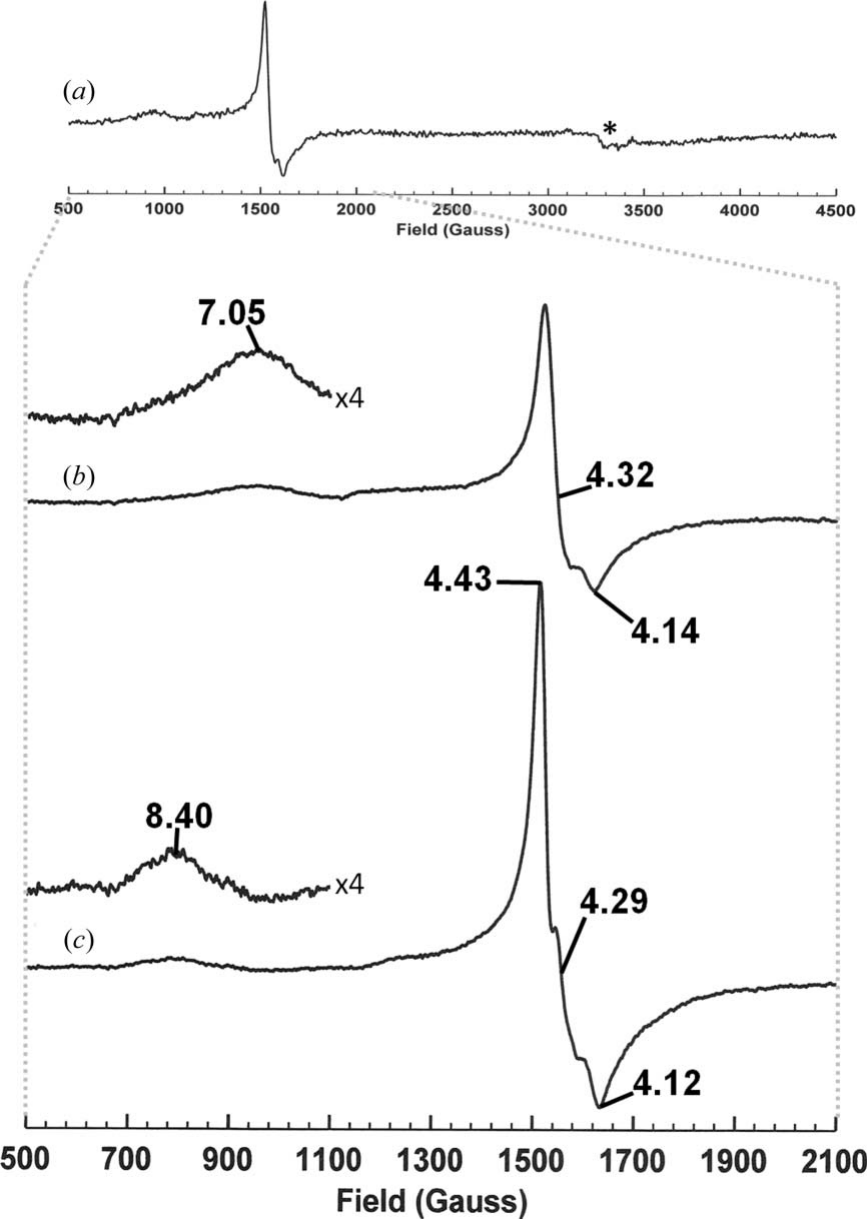
X-band EPR spectra of DAD. (*a*) 4000 G field sweep (an asterisk indicates trace Cu^2+^ contamination). (*b*) A sweep showing the region from 500 to 2100 G, with *g* values for major lines indicated. (*c*) DAD with a fivefold molar excess of substrate 2,4′-DHAP added, with *g* values for major lines indicated; vertical and horizontal scales are as in (*b*). Experimental parameters: microwave power 0.5 mW, modulation amplitude 5 G, modulation frequency 100 kHz, temperature 10 K; (*b*) and (*c*) are the result of four co-added scans.

The X-band EPR spectrum of DAD obtained over a wide field range (Fig. 6 ▶ *a*) shows significant lines between 600 and 1700 G and little else. Concentrating on this field range reveals an axial line with *g*_⊥_ = 4.32 and *g*_||_ = 4.14 together with a broader and weaker line centred at *g* = 7.05 (Fig. 6 ▶ *b*). The lines at *g* ≃ 4 arise from the middle doublet of an *S* = 5/2 high-spin ferric ion with zero field splitting E/D = 1/3 (rhombic), while the broader line at *g* ≃ 7 arises from the upper and low doublets of the same spin system. This spin system accounts for only one iron per 6–8% of the protein (based on integration relative to standard samples of ferric ion solutions), showing the protein is mainly ferrous as isolated. Adventitious high-spin ferric ions found in preparations of some proteins are associated with an isotropic spectrum at *g* = 4.26 distinct from that observed here. The addition of the substrate 2,4′-DHAP to DAD gives rise to an increase in the intensity of the spectrum around *g* = 4 and a rhombic splitting, *g*_1_ = 4.43, *g*_2_ = 4.29 and *g*_3_ = 4.12 (Fig. 6 ▶ *c*). Furthermore, the broad feature at *g* = 7.05 sharpens and moves downfield to *g* = 8.40. These observations support the assertion that the iron observed by EPR is bound at the active site of DAD since its environment is sensitive to substrate binding.

### 3.6. Modelling bound substrate   

**Figure 7:**
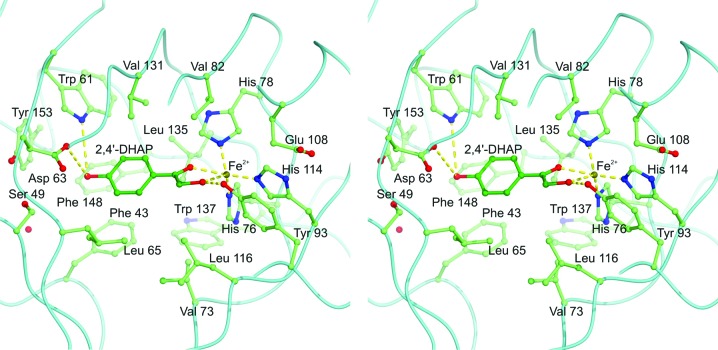
A stereoview of a model of the aromatic substrate 2,4′-DHAP in the active site. The 2,4′-DHAP (shown in dark green) occupies the positions of the putative glycerol and carbonate. The phenolic –OH group interacts with Asp63 and Trp61 by hydrogen bonds and the α-­hydroxyketone group of 2,4′-DHAP interacts with the active-site iron by dative bonding and with the side chain of Tyr93 by a hydrogen bond. Note that the side chain of Phe129 which, like Phe43, interacts with the substrate by ring stacking has been omitted from the foreground for clarity.

In order to model the binding of the aromatic substrate to the enzyme, a 2,4′-DHAP molecule was fitted to the active site such that its α-hydroxyketone group was in close proximity to the iron. In order for both O atoms of the α-hydroxyketone to bind the metal ion datively, it is necessary to displace the putative carbonate group. Interestingly, the α-hydroxyketone group of the substrate provides an excellent fit for the electron density of the putative carbonate group, and it is possible to fit this group to the approximately planar density for the carbonate in two orientations. However, only one of these orientations allows the aromatic part of the substrate to make favourable interactions within the pocket where the electron density for the putative glycerol resides. In this orientation, the α-hydroxy group forms a hydrogen bond to the conserved residue Tyr93 close to the iron (see Fig. 7 ▶). This model of the bound substrate has many satisfactory features such as the aromatic ring being stacked approximately between the conserved side chains of Phe43 and Phe129 and its phenolic –OH group being hydrogen-bonded to the conserved side chains of Trp61 and Asp63 (Fig. 7 ▶). The importance of these hydrogen bonds is corroborated by the observation that a substrate analogue lacking the phenol –OH group (2-hydroxyacetophenone) is turned over at 5% of the rate of the normal substrate (Enya *et al.*, 2012 ▶). Indeed, this model of substrate binding provides a reasonably satisfactory fit to the electron density for both the putative glycerol and carbonate molecules in the binding pocket, although not as good. Whilst there is no reason to believe that 2,4′-DHAP would be present in sufficient quantities in *E. coli* grown in LB medium, the quality of the fit does suggest that the enzyme may have acquired a molecule with some similarity to 2,4′-DHAP and this has co-purified and co-crystallized with it.

Another interesting feature of this model is that it allows us to predict where the dioxygen substrate will bind to the enzyme. As shown in Fig. 7 ▶, the two O atoms of the 2,4′-DHAP α-hydroxyketone group and the imidazole N atoms of histidines 78 and 114 act as equatorial ligands for the active-site iron. In contrast, the imidazole of His76 provides an axial iron ligand binding the metal from below. This arrangement leaves some space for an axial ligand to bind above the iron (in the view shown in Fig. 7 ▶), which is unoccupied in the X-ray structure. What is very interesting is that this empty pocket actually has a small but significant feature of positive difference density (2.5 r.m.s.) which extends from the iron itself towards Glu108 (Fig. 5 ▶ *c*). This finding led us to build a dioxygen into the model at this position and refine it with the X-ray data. However, the resulting map was rather un­satisfactory in this region, suggesting that if oxygen is present in the crystal it must have low occupancy. Nonetheless, it is an ideal position at which oxygen could bind from structural and mechanistic viewpoints. The glutamate (108) which would interact with an oxygen bound here appears to be completely invariant (see Fig. 3 ▶), suggesting that it has an important function. Glu108 effectively shields the active-site iron from solvent surrounding the enzyme and the electron-density map indicates that it has some flexibility. Both these observations suggest that Glu108 may play important roles in allowing dioxygen to gain access to the active-site iron and in binding an oxygen molecule at this position.

## 4. Discussion   

We report the first-in-class structure of the enzyme 2,4′-dihydroxyacetophenone dioxygenase (DAD) from *Alcaligenes* sp. 4HAP. The enzyme is a very unusual dioxygenase in that it is involved in C—C bond cleavage in a substituent of the aromatic ring rather than in the ring itself. The structure refined at 2.2 Å resolution establishes that the enzyme adopts a cupin fold forming tightly associated dimers with a pronounced hydrophobic interface between the monomers. The catalytic iron is coordinated by three histidine residues (76, 78 and 114) within a buried active-site cavity. The iron is tightly coordinated by an additional ligand which we have putatively interpreted as a carbonate group. Dissolved CO_2_ is present in the form of carbonic acid, bicarbonate and carbonate at a collective level of ∼10 m*M* within cells since they form the predominant biological buffer system in living organisms. Carbonate is also a commonly found ligand in structures of nonhaem iron proteins. In attempting to interpret the electron density for this group, which has a pronounced planar, triangular appearance, several ligands were built in and refined, and carbonate was found to give the most satisfactory fit. Another possibility is the product of the reaction, formate, although refinement of this as a possible ligand resulted in unexplained positive difference density which was accounted for very well by the additional O atom that is present in carbonate. However, since both formate and acetate are major fermentation products in *E. coli* (see, for example, Kirkpatrick *et al.*, 2001 ▶), we consider these to be strong possibilities for the unknown iron ligand, although both would be unable to hydrogen-bond to the putative glycerol moiety as well as carbonate. Interestingly, this ligand is located in a position which is highly likely to be occupied by the α-hydroxyketone group of the bound aromatic substrate during catalysis. Modelling of the substrate molecule in the active-site cavity indicates that it will interact with many conserved amino acids in the predominantly hydrophobic active-site pocket, where it most likely undergoes peroxide radical-mediated heterolysis.

While active DAD contains predominantly ferrous iron, the presence of ferric iron at the active site in approximately 6–8% of the enzyme provides a very convenient EPR probe. This approach has been employed in the study of cysteine dioxygenase (CDO), a mononuclear ferrous iron-containing cupin related to DAD which also has a three-His iron-binding site (Crawford *et al.*, 2011 ▶). EPR of ferric CDO exhibited an isotropic line at *g* = 4.3 with a broader, less intense line at *g* = 8.2. On binding the substrate cysteine, the line at *g* = 4.3 became more intense and developed a rhombic anisotropy, while the line at *g* = 8.2 moved downfield to *g* = 9.7. Thus, the behaviour of DAD and CDO are qualitatively the same, suggesting that iron ligation and substrate-binding geometry are very similar in the two enzymes. The only other cupin dioxygenase with three-His iron coordination that we are aware of is gentisate 1,2-dioxygenase (Chen *et al.*, 2008 ▶).

**Figure 8:**
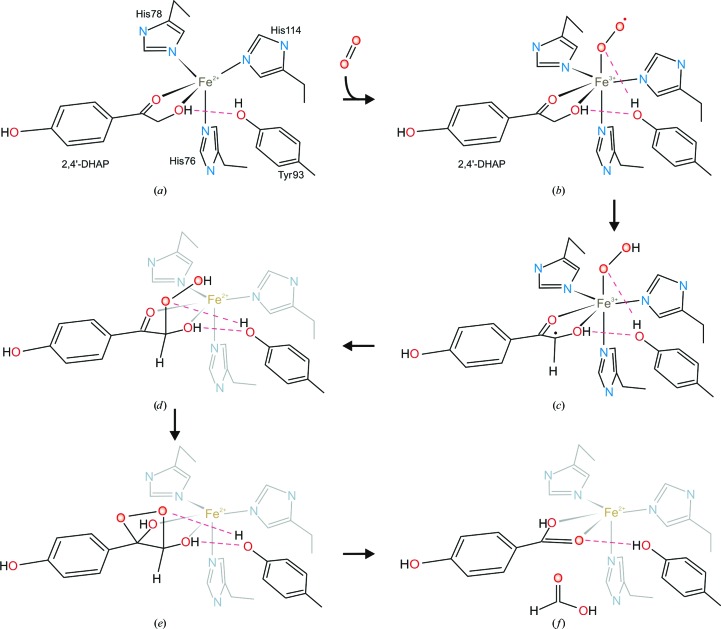
The catalytic mechanism of DAD. (*a*) shows the substrate complex, as in Fig. 7 ▶, and the following parts (*b*–*f*) indicate the steps of the reaction proposed by Paria *et al.* (2012 ▶) which is consistent with our experimentally determined structure. For clarity, steps (*d*–*f*) show the active-site histidines and the iron greyed-out since they are in the background in this view. The scheme emphasizes the key role played by the –OH group of Tyr93 in the stabilizing the intermediates.

We used the model of bound substrate described in the previous section as a starting point for investigating the mechanism of the enzyme, using a scheme that was proposed recently from detailed structural and spectroscopic studies of biomimetic model compounds by Paria *et al.* (2012 ▶) as a framework. Their studies involved the synthesis of an iron(II)-tris-pyrazole borate compound which mimics the three active-site histidines of DAD (76, 78 and 114) bound to iron. This compound was then complexed with an analogue of the substrate, 2-hydroxyacetophenone, and the crystal structure was determined (Paria *et al.*, 2012 ▶). Their structure showed that this model complex has many of the key features that are expected to occur at the active site of the enzyme, such as direct coordination of the iron by three pyrazole-ring N atoms and both O atoms of the α-hydroxyketone group. Intriguingly, this compound was found to react with oxygen, yielding the expected products benzoic and formic acid. Paria *et al.* (2012 ▶) propose that oxygen binding leads to the formation of an iron(III)-superoxide species (Fig. 8 ▶ *b*), which abstracts a hydrogen from the α-carbon of the hydroxyketone group, giving an iron(III)-hydroperoxide intermediate (Fig. 8 ▶ *c*). A radical rebound mechanism (Groves *et al.*, 1978 ▶) then leads to hydroperoxylation of the substrate, giving an intermediate (Fig. 8 ▶ *d*), and the subsequent C—C bond cleavage of this intermediate, potentially *via* a dioxacyclic intermediate (Fig. 8 ▶ *e*), leads to the formation of both carboxylic acid products (Fig. 8 ▶ *f*). Our structure indicates that these peroxy intermediates reside in a pocket formed by the side chains of Val82 and Phe129, with the phenolic –OH group of Tyr93 playing a key stabilizing role in the mechanism. It is notable that these three residues are invariant in the alignment shown in Fig. 3 ▶, as is Glu108, which we suggest may play a role in shuttling oxygen into the active site. Thus, the work reported here offers the first detailed structural rationale for the catalytic properties of DAD and provides a basis for studying substrate and inhibitor binding, as well as for designing site-directed mutants to test the catalytic roles of residues at the active site and for theoretical studies of the mechanism.

## Supplementary Material

PDB reference: 2,4′-dihydroxyacetophenone dioxygenase, 4p9g

## Figures and Tables

**Table 1:** Data-collection and processing statistics for 2,4-dihydroxyacetophenone dioxygenase from *Alcaligenes* sp. 4HAP Values in parentheses are for the outer resolution shell.

Beamline	BM14, ESRF
Wavelength ()	1.072
Space group	*P*6_5_22
Unit-cell parameters	
*a* = *b* ()	82.6
*c* ()	114.0
Mosaic spread ()	0.18
Resolution ()	57.02.2 (2.32.2)
*R*_merge_ † (%)	13.6 (61.9)
*R*_meas_ ‡ (%)	14.3 (66.8)
CC_1/2_ § (%)	99.6 (80.1)
Completeness (%)	86.3 (49.4)
Average *I*/(*I*)	12.6 (3.1)
Multiplicity	9.8 (7.1)
No. of observed reflections	103761 (5973)
No. of unique reflections	10549 (836)
Wilson plot *B* factor (^2^)	29.2
Solvent content (%)	56.2
No. of molecules per asymmetric unit	1
*R* factor (%)	17.3
Free *R* factor (%)	21.8
R.m.s.d., bond lengths ()	0.02
R.m.s.d., bond angles ()	1.9
No. of reflections in working set	10021
No. of reflections in test set	512
Mean protein *B* factor (^2^)	29.7

† *R*_merge_ = 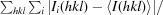 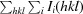.

‡ *R*_meas_ = 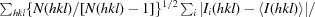 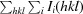, where *I_i_*(*hkl*) is the mean intensity of the *N*(*hkl*) observations *I_i_*(*hkl*) of each unique reflection *hkl* after scaling.

§ CC_1/2_ is the half-set correlation coefficient as described by Karplus Diederichs (2012 ▶).
